# Contribution of HIV/AIDS-Related Human and Social Sciences Research to a Better Understanding of the Challenges of Hepatitis B Prevention, Diagnosis and Care

**DOI:** 10.3390/microorganisms9061166

**Published:** 2021-05-28

**Authors:** Charlotte Bauquier, Marie Préau

**Affiliations:** Research Group in Social Psychology (GRePS UR 4163), University Lyon 2, 69676 Bron, France; marie.preau@univ-lyon2.fr

**Keywords:** HIV/AIDS-related HSS, challenges of hepatitis B, screening, retention in care, quality of life

## Abstract

Recent scientific advances in hepatitis B virus research hint at the possibility of finding a cure in the medium term. In this context, the characterization of infected persons constitutes a major public health issue in terms of implementing adapted screening and prevention strategies. Overcoming the current challenges national health systems face in hepatitis B diagnosis is essential if the World Health Organization’s target of treating 80% of infected patients by 2030 is to be reached. These challenges reflect those previously faced in the fight against HIV/AIDS. Using the knowledge produced to date in Human and Social Sciences research in the fight against HIV/AIDS, we propose avenues of reflection to support and guide the development of research in the diagnosis of hepatitis B infection. More specifically, we present theoretical, methodological and epistemological considerations for how HSS research can be optimized in the following three HBV diagnosis-related areas: (i) access to screening; (ii) retention in care; and (iii) the integration of quality of life measurement in clinical trials.

## 1. Introduction

Hepatitis B virus (HBV) is responsible for half of the deaths from viral hepatitis worldwide, particularly in eastern Asia and sub-Saharan Africa [1]. Recent scientific advances in HBV research hint at the possibility of finding a cure in the medium term. In this context, the characterization of infected persons constitutes a major public health issue in terms of implementing adapted screening and prevention strategies [2]. Early screening and early diagnosis are effective secondary prevention tools in the fight against epidemics. They constitute a gateway to prevention and treatment services. Accordingly, the sooner people with HBV infection are diagnosed, the sooner they receive the necessary care to prevent—or at least delay—the progression of liver disease, cirrhosis and liver cancer. Prevention also covers the notion of transmission to others, and HBV screening provides healthcare professionals the possibility to implement comprehensive, tailored transmission prevention strategies suited to individuals and to populations. This includes informing persons about risky behaviors that foster the transmission of the virus, and offering them prevention and harm reduction materials such as sterile syringes. The screening/diagnosis stage brings up many issues for HBV-infected people: quality of life, risk of stigmatization, acceptability of and adherence to new care strategies. Responding to these issues is fundamental if the World Health Organization’s (WHO) objectives for 2030 are to be met, specifically, that 90% of infected people will be aware of their HBV serological status, and that 80% of eligible HBV-infected patients will be treated [1]. These challenges are very reminiscent of those encountered in the fight against HIV/AIDS over the past ten years, and reflect the UNAIDS 90-90-90 HIV targets for 2020 which were set out in 2014 (90% of people living with HIV (PLHIV) screened, 90% of PLHIV on treatment, and 90% of PLHIV with viral load suppression) [3]. Using the knowledge produced in Human and Social Sciences (HSS) research in the fight against HIV/AIDS—which made it possible to build solid research protocols and the acquisition of robust scientific data—we propose avenues of reflection to support and guide the development of HSS research in the diagnosis of HBV infection. Specifically, the present article presents three perspectives which reflect different points in the HBV infection care cascade: (i) access to screening, (ii) retention in care of people living with chronic HBV infection (PLHBV), and (iii) the integration of quality of life measurement in clinical trials and cohort follow-ups.

## 2. Prevention of Hepatitis B Infection: Access to Screening

The proportion of HBV-positive people in metropolitan France (i.e., mainland and Corsica) unaware of their status is estimated at 18%. This data is representative of a high-income country however, and the WHO estimates that the proportion of people worldwide who are unaware of their infection is approximately 95% (much higher than estimates for PLHIV [2]), a figure which underlines the huge public health importance of greater research in access to HBV screening. Like HIV, HBV infection is most often asymptomatic, leading to late diagnosis. Patients only consult for symptoms related to chronicity and to diseases which develop as a result of infection (e.g., liver disease, cirrhosis and liver cancer) which current treatments cannot completely cure. Spontaneous individual HBV screening is recommended at least once in a person’s life. Regular screening is recommended for pregnant women and people in high-risk contexts (such as those practicing unprotected sex, and travelers to geographic areas with a high prevalence) [4]. In adults, a large proportion of HBV infections occur through sexual relations [4,5]. Several studies have focused on the social determinants associated with hepatitis B vaccination [6,7], and on ways to limit mother-to-child transmission [8,9]. Many other studies have investigated the implementation of screening programs, especially those related to public health policy [2,10]. However, few studies to date have explored the facilitators and barriers to HBV screening from a psychosocial perspective. Unlike HBV, the psychosocial issues surrounding screening have been widely documented in the field of HIV research. One example is perceived risk, a concept based on several theoretical foundations from several fields of research. Assessing perceived risk has been shown to be a useful and valid scientific tool for a greater understanding of the factors involved in individuals’ and groups of individuals’ implementations (or not) of HIV infection/transmission prevention behaviors.

With regard to the decision to go for HIV screening, two key elements have been highlighted in psychosocial studies, specifically (i) the perception of individual and collective risks, and (ii) the stigma associated with the diagnosis of the disease. We hypothesize that these two elements also impact people’s decisions to go for HBV screening. At an individual level, the perceived severity of the infection as well as one’s own perceived vulnerability to the risk of infection, impact prevention behaviors. These two elements are taken into account in several sociocognitive models, including the Health Belief Model and the protection motivation theory. They are also considered in comparative optimism, which is the belief that more positive events will happen to oneself than to others [11,12]. These sociocognitive models and comparative optimism are rooted in motivational and cognitive explanations, and accordingly, form part of a very individual-centered approach to understanding prevention behaviors. However, they struggle to correctly predict certain prevention and harm reduction practices, as they do not—or only marginally—take into account contextual, affective and relational issues. Studies conducted in the field of HIV illustrate this shortcoming perfectly [13,14,15,16]. Implementing prevention behaviors is a complex phenomenon which brings into play several dimensions (e.g., affect), each of which acts at various levels (e.g., sexual practices, risk perception, identity and group culture, global relationship to health or even the notion of pleasure). Accordingly, by developing a comprehensive approach that takes into account the meaning which individual members of a given social group give to their heath behaviors, it becomes possible to understand risk perception in a much more detailed and complete way. When faced with epidemic risks, norms are built within the group. This makes it possible to reduce individual uncertainty in the face of danger, and to regulate prevention practices by orienting the behavior of individual members of the group. This is done in particular by attributing a moral dimension to them (e.g., what is good, what is acceptable to do or not) [17]. Consequently, research that is too focused on individuals and that decontextualizes individuals from groups who make sense for them, leads to a less than optimal understanding of the issues involved. Analysis at the group level is also essential to understand the second element highlighted above in terms of people’s decisions to go for HIV screening or not, specifically the phenomenon of stigma associated with the diagnosis of the disease. The fear of being stigmatized can lead to risk-taking, for example, not going for screening not seeking care after positive diagnosis [18,19,20], and not disclosing one’s HIV status with loved ones or potential sexual partners [17,21,22].

It is in this context that the importance of the need to implement combination HIV prevention [23] takes on its full meaning, through a wide arsenal of prevention and harm-reduction tools, including making self-tests available, and offering screening in association-based and community spaces [24,25,26]. The logic of implementing combination prevention for HIV is absolutely valid for the field of hepatitis B prevention, where many inequalities in access to care—especially for vaccination—persist [27]. Combination prevention is essential to adapt screening systems (e.g., outreach screening) to the specific experiences of populations affected by HBV infection. Accordingly, it is vital to develop research aimed at better understanding these experiences within the diverse contexts concerned.

## 3. How to Guarantee Retention in Care of People Diagnosed with HBV?

HBV infection can become chronic (defined as the presence of virus surface antigen six months after infection [28]). Specifically, 5 to 10% infections in adults can become chronic and progress to cirrhosis or hepatocellular carcinoma (i.e., liver cancer). This figure rises to 90% in infants. While only 10 to 40% of people diagnosed need to have treatment—most often with first-line oral therapy—all must follow the HBV care pathway so that disease progression can be monitored and potential liver damage prevented. As no cure exists yet, current treatment focuses on slowing progression to cirrhosis and HCC, by blocking the replication of the virus [29]. Based on the results of a systematic review by Lieveld et al. [30], overall adherence to HBV treatment is quite good. However, since treatment adherence is a fundamental factor in the success or failure of virologic control, the same authors stressed the importance for clinicians to identify patients with poor adherence. Once again, research in the field of HIV/AIDS has widely documented the psychosocial processes involved in treatment adherence, in particular after the advent of ‘triple therapy’ treatment. HSS researchers were called very early on to explore avenues to ensure optimal adherence to treatment in PLHIV, especially to multiple-therapy treatment [31]. Moreover, the most recent biomedical data underline the public health importance of good adherence in terms of prevention and limiting the spread of the HIV at the population level. More specifically, data show that an undetectable viral load and a zero risk of transmission can be accomplished when people adhere fully to their treatment. Various objective and subjective tools exist to assess adherence. One example of the former is to count the number of pills taken over a certain period. An example of the latter is to directly ask patients about their adherence using a questionnaire. One such questionnaire is the CEAT-HIV, which has been validated in several countries, including Brazil, Portugal and Romania [32]).

Identifying the people most at risk of not adhering to HBV treatment is essential in order to provide them with support as early as possible in the care process. This is how the results from early research on treatment adherence in the field of HIV were of great importance, as they highlighted the need for an empathic, non-predictive approach to assessing treatment adherence [33]. In this approach, it is essential to have subjective patient assessments of their adherence, combined with both validated and subjective assessments of their experiences with the disease and its treatment [34]. More specifically, patients’ representations of HIV [35], their experience with treatment, and the psychosocial issues involved in living with the disease, are all crucial to acquire an in-depth understanding of the determinants of adherence [36]. Accordingly, it is essential to view adherence as a dynamic process that evolves over time [37,38]. In terms of the determinants of treatment adherence, the risk of stigmatization—as highlighted above—is one such determinant to take into account when trying to understand an infected person’s decision not to seek care. Being able to implement support systems for HIV-infected people while respecting their choice to say or not to disclose their HIV status, has been highlighted as an essential element in the fight against the epidemic, from a global public health perspective [39]. With this in mind, understanding the factors at play when a person infected with HBV decides whether or not to disclose their HBV serological status, and to whom, presents itself as a very interesting avenue for exploration in HSS research, in terms of suggesting tailored support systems. Such studies would also help advocate the integration of diagnosed individuals into the HBV care pathway and, where applicable, their adherence to treatment.

## 4. Clinical Trials and Cohort Monitoring: Integrating Quality of Life

**Figure 1:**
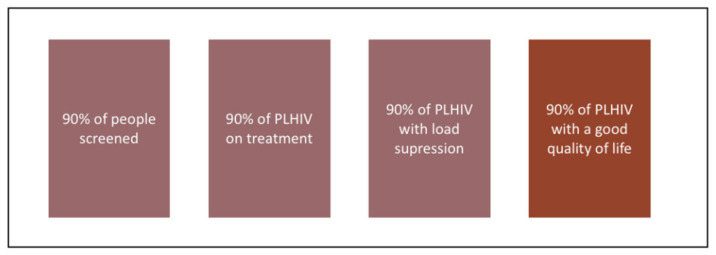
UNAIDS 90-90-90 targets and the 4th target added by Lazarus et al. [54].

Measuring quality of life (QoL) is a very useful way to understand people’s experiences with and the effects of treatments, in order to anticipate them as well as possible for future patients. QoL is a concept used in several research disciplines which is capable of objectifying what is by definition subjective, namely the experiences of the people concerned. Assessing QoL means putting the patient at the heart of the care process [40]. A multidimensional concept, it integrates the physical, psychological and social dimensions of life. Several psychosocial factors (environment, adverse effects, societal norms, body image, stigma, etc.) can influence a patient’s QoL [41]. The concept of treatment adherence is strongly and positively linked to that of QoL [42]. As a result, many authors have evaluated the QoL of people living with acute or chronic HBV, whether on treatment or not. Their results highlight that the more advanced the disease, the more multiple dimensions of QoL are negatively affected [43,44,45,46]. As pointed out in the introduction section above, recent results from HBV clinical treatment trials suggest the possibility of a cure for the disease in the medium term. In this perspective, research in the field of HIV has emphasized the need to take into account the QoL of people who volunteer to participant in clinical trials and in cohort studies [40,47]. As is the case for PLHBV, several studies have investigated the QoL of PLHIV [48,49,50,51], in particular their emotional and sexual QoL [52,53]. For Lazarus et al. [54], a good QoL for PLHIV must become a care objective in itself (Figure 1). This is especially important given that current research tends to show that even in PLHIV with HIV viral load suppression, their QoL is lower than that measured in the general population [54]. This novel objective is part of a vision where treatments for infectious diseases not only aim to suppress the viral load, but also seek to promote the well-being and QoL of PLHIV in all its dimensions. As a result, measuring QoL is essential in any work on these treatments. The challenges encountered when trying to evaluate QoL—which, by definition, is a subjective measurement—must not hinder its evaluation. To ensure this, it is important to increase stakeholders’ awareness that fully validated and adaptable QoL measurement tools already exist both for HIV [55] and HBV [56].

However, irrespective of how it is measured, the determinants of QoL must also be understood by integrating psychosocial dimensions [40]. This is essential if we are to really understand the experience of patients, especially as psychosocial dimensions help highlight issues of stigmatization [57] and mental health [58,59] which can significantly affect QoL of PLHIV and PLHBV. Similarly, as QoL is a dynamic concept that evolves over the course of an individual’s lifetime (see above) where various events occur (diagnosis, side effects of treatments, interruption of professional activity, etc.), it seems essential to ensure that it is measured using a longitudinal approach, as the adaptive coping processes individuals implement over time can then be taken into account. This approach is the basis of response-shift theory [60,61], and could be particularly useful in the field of HBV.

## 5. Conclusions

In this paper, we have argued and proposed three possible avenues for HSS research in the field of hepatitis B, based on lessons learned from research on HIV/AIDS over the past twenty years, and taking into account the WHO’s HBV objectives for 2030. Firstly, improving access to screening is essential to promote early diagnosis. In order to try and reach this objective, research should explore the individuals’ perceptions and representations of risks by taking into account their group and social insertions to apprehend prevention practices more accurately. Secondly, it is important to maintain an optimal treatment adherence for PLHBV and identify patients who are more likely to not cure themselves. For this, an empathic approach to adherence, as well as the study of factors at stake when a person infected with HBV decides whether or not to disclose their HBV serological status could facilitate the integration of individuals into the care pathway. Thirdly, it seems essential to make the measure of the quality of life of PLHBV a real criterion for assessing clinical trials and cohorts monitoring. We believe that the implementation of multidisciplinary HBV research projects which combine HSS, biomedical sciences and community-based participatory approaches, are essential, both in terms of scientific innovation and social change, especially as such projects have already proven their worth in the fight against HIV/AIDS [62].

## Data Availability

Not applicable.
